# *Leishmania infantum* infection in *Phlebotomus perniciosus* and *Phlebotomus perfiliewi* (Diptera, Psychodidae) and their abundance in central Italy

**DOI:** 10.1186/s13071-026-07477-z

**Published:** 2026-06-04

**Authors:** Irene Del Lesto, Manuela Scarpulla, Claudio De Liberato, Adele Magliano, Sara Greco, Laura Salvato, Giovanni Marafini, Arianna Ermenegildi, Valeria Carioti, Matilde Chiavacci, Carola Cartolano, Rosaria Cacace, Barbara Rita Porchia, Alessandro Millo, Federico Romiti

**Affiliations:** 1 Istituto Zooprofilattico Sperimentale del Lazio e della Toscana, M. Aleandri, Via Appia Nuova, 1411, 00178 Rome, Italy; 2 Dipartimento Prevenzione e Sanità Animale, Azienda Usl Roma 5, Piazza Massimo, 1, 00019 Tivoli, Italy; 3 Dipartimento Prevenzione UF SPVSA Azienda USL Toscana sudest, Viale Cimabue, 109, 58100 Grosseto, Italy; 4 Regione Toscana, Direzione Diritti di Cittadinanza e Coesione Sociale Settore Prevenzione Collettiva, Via Taddeo Alderotti, 26/N, 50193 Florence, Italy

**Keywords:** Sand flies, Leishmaniasis, Vector surveillance, Phenology, Epidemiology

## Abstract

**Background:**

Leishmaniasis caused by the parasitic protozoan *Leishmania infantum* is endemic in central and southern Italy, but systematic vector surveillance has historically been lacking. Following human cases of visceral leishmaniasis reported in the Lazio and Tuscany regions of Italy, we implemented a surveillance system aimed at describing the occurrence, seasonal dynamics and infection rates of the main vector species in these regions, consequently identifying periods and areas at higher risk of transmission.

**Methods:**

Sand flies were collected with CDC-CO_2_ light traps from April to November 2023 at 75 rural and suburban farms and at three additional sites close to reported human leishmaniasis cases in urban areas. Traps were operated overnight at 1- or 2-week intervals, and the collected sand flies were sorted, counted and identified morphologically. Sites where sand flies were detected were characterised using bioclimatic variables and altitude, and then grouped into climate clusters using* k*-means clustering. Seasonal activity was analysed by fitting Gaussian generalised linear models with a negative binomial distribution to the abundance data. Pools of female sand flies were tested for *L. infantum* by real-time PCR, and infection rates were estimated per site and sampling date.

**Results:**

A total of approximately 96,333 sand flies were captured in 40 of the 78 collection sites. Of the 13 provinces monitored, nine were positive for sand flies. Two species of sand flies were identified: *Phlebotomus perfiliewi* (76% of identified specimens) and *P. perniciosus* (24%), which co-occurred in most provinces. Three distinct climate clusters were defined, mainly separating coastal low-altitude areas from more inland, hilly sectors. Across clusters, adult sand fly activity peaked in early August, with longer activity periods and higher predicted abundances in coastal and low-altitude areas dominated by *P. perfiliewi*. *Leishmania infantum* DNA was detected in sand fly pools from eight sites in six provinces, with infected sand flies present from late June to mid-October. The highest infection estimates occurred at high-abundance rural foci dominated by *P. perfiliewi* and at hilly sites where *P. perniciosus* was more abundant.

**Conclusions:**

Both *P. perniciosus* and *P. perfiliewi* contribute significantly to *L. infantum* circulation in central Italy. The areas at risk, seasonal vector dynamics and infection rates identified in this study provide essential information for the timing and targeting of veterinary and public health interventions and awareness campaigns.

**Graphical Abstract:**

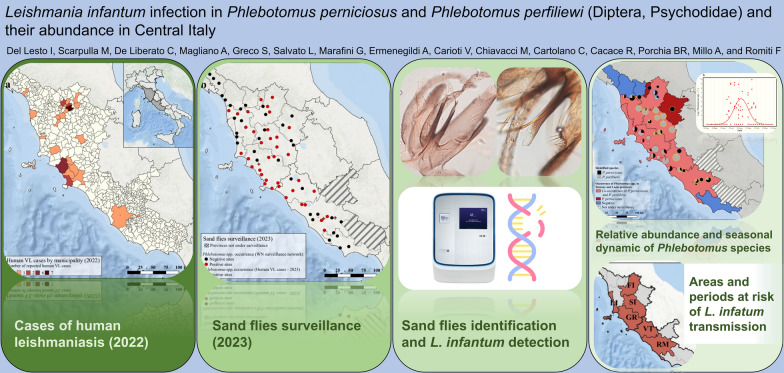

## Background

Sand flies of the subfamily Phlebotominae (Diptera: Psychodidae) are haematophagous dipterans of medical and veterinary relevance due to their role as vectors of several pathogens, including protozoa, bacteria and viruses, affecting both human and animal populations [1].

Among the pathogens transmitted by sand flies, parasitic protozoans of the genus *Leishmania* (Kinetoplastida: Trypanosomatidae) are responsible for leishmaniasis, which is widely distributed in tropical and subtropical areas of North and South America, Europe, Asia and Africa [2]. Human leishmaniasis (HL) occurs in three forms: visceral (VL), cutaneous (CL) and mucocutaneous (ML). VL and CL, as well as canine leishmaniasis (CanL), are distributed worldwide, with the exception of Oceania and Antarctica [3, 4], while the ML form is endemic in Latin America, with sporadic cases reported in Europe [5]. On a global scale, more than 1 billion people live in areas endemic for leishmaniasis and are at risk of infection, with an estimated 30,000 new cases of VL and more than 1 million new cases of CL occurring annually [6].

In the European Union (EU) and neighbouring countries, VL and CL are mainly caused by four species: *Leishmania infantum* and *Leishmania donovani* sensu stricto (*Leishmania donovani* s.s.), both belonging to the *L. donovani* complex, *Leishmania major* and *Leishmania tropica* [7, 8]. *Leishmania infantum* is found in several southern European countries, while *L. donovani *s.s. has only been described in Cyprus and Turkey and *L. major* and *L. tropica* are restricted to northern Africa and some parts of the Caucasus [9]. Leishmaniasis, which is endemic in several countries of the Southern and Eastern Mediterranean Basin, is considered to be an emerging disease in temperate areas of central Europe [10, 11]. In addition, a notable northern expansion in the geographical range of two relevant *Leishmania* vectors, i.e. *Phlebotomus perniciosus* and *Phlebotomus perfiliewi*, has been predicted in Europe [12, 13] and recorded in many EU countries [14–18].

Central and southern Italian regions are endemic for HL and CanL caused by *L. infantum* [19–21]. The primary vector, *P. perniciosus*, is particularly abundant in the Tyrrhenian and southern regions of Italy [22, 23]. Although considered a less competent vector, *P. perfiliewi*, which is abundant in central Italy, has been associated with *L. infantum* transmission in southern Italy [24, 25]. Moreover, Italy is the European country with the highest seroprevalence of CanL and has recorded an increase in the number of VL cases in humans [22, 26]. Despite this, vector-based surveillance, aimed at highlighting periods and areas at high risk of transmission, is still lacking.

Data on the seasonal dynamics of sand fly species originate from research studies, but these rarely investigate the phenology of both vector and parasite [27–29]. Investigating the infection rate in *Phlebotomus* vectors and identifying periods and areas characterised by a high intensity of *Leishmania* transmission could provide useful data for focusing prevention campaigns.

 Following human cases reported in 2022 and 2023 in the Lazio and Tuscany regions of central Italy by the local health authorities [30], we carried out an entomological surveillance to collect data on the occurrence, abundance and seasonality of *Phlebotomus* spp. in the two regions and to determine the rate of sand fly infection with *L. infantum*.

## Methods

### Entomological surveillance

**Fig. 1:**
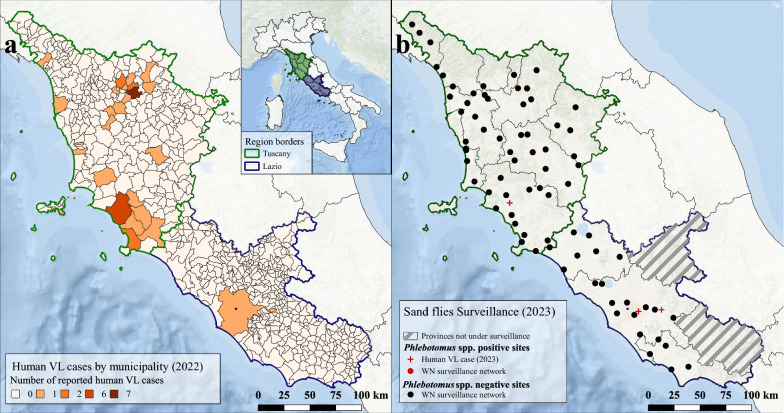
**a **Spatial distribution of human leishmaniosis cases in the Tuscany and Lazio regions in 2022 pooled by municipality, using data from local health authorities. **b** Spatial distribution of the sampling sites where Phlebotominae surveillance was carried out, with the West Nile surveillance catches denoted by circles and ad-hoc catches denoted by red crosses. VL, Visceral leishmaniasis; WN, West Nile

Following the reports of human cases of VL from autumn 2022 to March 2023 in the Lazio and Tuscany regions of Italy (Fig. 1a), we carried out a sand fly surveillance study using CDC-CO_2_ light traps (Fig. 1b), which are commonly used for Phlebotominae studies [31–33]. Sampling relied on the West Nile surveillance network, according to the National Plan for Prevention, Surveillance and Response to Arboviroses (NPA) 2020–2025 [34]. Following the guidelines of the NPA, the territory of the two regions was divided into 20 × 20-km cells, with an average altitude of < 600 m a.s.l., and then a CDC-CO_2_ light trap was set in each cell. Each CDC-CO_2_ light trap was operated overnight at 2-week intervals in the period April–November. This results in the placing of 75 CDC-CO_2_ light traps in cattle, horse or pig farms in rural and suburban areas, outside the stables near run-down premises.

Following the report of VL cases during the spring–summer 2023 by the Local Health Authority, we set three additional CDC-CO_2_ light traps in the Tuscany (*N* = 1) and Lazio regions (*N* = 2) (Fig. 1b). In these cases, the traps operated overnight on a weekly or two-weekly basis, depending on the availability of Health Services technicians, starting from the notification of each case and operating for at least 2 months.

Captured sand flies were analysed under the stereomicroscope to quantify the abundance of males and females. To assess species relative abundance in each sampling site, a subsample of males (minimum = 1, maximum = 20) in each catch was clarified in lactophenol for at least 24 h and morphologically identified by analysing hypopygium shape [35]. Females were not used for species identification to avoid potential interference of lactophenol clarification with subsequent molecular analysis.

For catches not exceeding 500 specimens, all individuals were sexed and counted. For abundant catches (14 catches exceeded 500 individuals), weight was used as a proxy for the number of individuals by weighing subsamples of a known number, and sex ratio was estimated analysing 100 individuals.

### *Leishmania infantum* testing

Female sand flies from the same catch (i.e. the same site and date of capture) were pooled in a single vial when at least three females were present and the total sand fly number did not exceed 500 individuals. For abundant catches, both males and females were pooled together in order to facilitate laboratory analyses. Pools of sand flies, with a maximum estimated number of 1000 individuals for abundant catches, were prepared and stored at − 20 °C until DNA extraction.

#### DNA extraction

Genomic DNA was extracted from sand fly pools using the QIAsymphony DSP Virus/Pathogen Kit (Qiagen, Hilden, Germany) and purified using the automated QIAsymphony SP nucleic acid purification instrument (Qiagen), following the manufacturer’s instructions.

Captured female phlebotomines were processed in pools of up to 1000 insects in a Class II biosafety cabinet. Each pool was incubated overnight in 2-ml Eppendorf tubes at 56 °C in an Eppendorf ThermoMixer C (Eppendorf, Hamburg, Germany) with the addition of a stainless-steel bead, 400 µl of ATL buffer (Qiagen) and 40 µl of proteinase K (PK). After incubation, the samples were first homogenised in a bead-mill homogeniser (TissueLyser II; Qiagen) at 30 Hz for 3 min, following which the tubes were centrifuged at 13,200 rpm for 10 min at 4 °C using a Heraeus Fresco 17R microcentrifuge (Thermo Fisher Scientific, Waltham, MA, USA). The resulting supernatant was subjected to automated DNA extraction, and the extracted DNA was eluted in 60 µl of Elution Buffer AE (Qiagen).

#### PCR amplification

The presence of *L. infantum* was determined using a real-time PCR assay targeting the conserved region of the minicircle kinetoplast DNA (kDNA) of the parasite [36]. The real-time PCR assay was performed in a 20-µl reaction volume containing 1 × TaqMan Master Mix (Applied Biosystems, Thermo Fisher Scientific), which includes buffer, MgCl_2_, dNTPs and Taq DNA polymerase, 500 nM of each primer [Q Leish-DOWN (5ʹ-AAAATGGCATTTTCGGGCC-3ʹ) and Q Leish-UP (5ʹ-GGCGTTCTGCGAAAACCG-3ʹ)], 200 nM of the fluorogenic probe (5ʹ-FAM-TGGGTGCAGAAATCCCGTTCA-3ʹ-BHQ1), 2 µl of extracted DNA and 1 × Exo IPC Mix and 1 × Exo IPC DNA as exogenous internal controls labelled with VIC™, according to the manufacturer’s instructions (TaqMan Exogenous Internal Positive Controls; Applied Biosystems, Thermo Fisher Scientific).

Thermal cycling conditions consisted of an initial denaturation at 95 °C for 10 min, followed by 40 cycles at 95 °C for 15 s and 60 °C for 1 min. Amplification was carried out using a QuantStudio 7 Flex system (Applied Biosystems, Thermo Fisher Scientific) on a 96-well plate, with fluorescence signal acquisition at 60 °C. Each PCR run included a positive control containing genomic *L. infantum* DNA and a negative control. Samples with cycle threshold (Ct) values ≤ 40 were considered to be positive for *L. infantum*.

### Statistical analysis

#### Clustering sites according to climate data

All sites positive for the presence of *Phlebotomus* spp. were clustered according to climate data and altitude to highlight any differences in sand fly adult activity among clusters. Climate data were obtained in raster format from the WorldClim database, at the highest available spatial resolution (under a kilometre per side of the raster cell in central Italy) [37]. The WorldClim database provides 19 bioclimatic variables (BIO) that represent historical means of climate data (1970–2000) on annual trends, seasonality and extreme or limiting climate factors (Table 1). Climate data and elevation above sea level data were downloaded using R software (version 4.2.2; R Foundation for Statistical Computing, Vienna, Austria), and mean values were calculated within a 1-km buffer around each sampling site, using the ‘raster’ and the ‘sf’ R packages [38–40].

**Table 1:** Summary of the climate variables used for the clustering analysis of Phlebotominae-positive sites

Climate variable code	Description
BIO 1	Annual mean temperature
BIO 2	Mean diurnal range: mean of monthly (max temp.—min temp.)
BIO 3	Isothermality (BIO 2/BIO 7) (× 100)
BIO 4	Temperature seasonality (standard deviation)
BIO 5	Max temperature of warmest month
BIO 6	Min temperature of coldest month
BIO 7	Temperature annual range (BIO 5 - BIO 6)
BIO 8	Mean temperature of wettest quarter
BIO 9	Mean temperature of driest quarter
BIO 10	Mean temperature of warmest quarter
BIO 11	Mean temperature of coldest quarter
BIO 12	Annual precipitation
BIO 13	Precipitation of wettest month
BIO 14	Precipitation of driest month
BIO 15	Precipitation seasonality (coefficient of variation)
BIO 16	Precipitation of wettest quarter
BIO 17	Precipitation of driest quarter
BIO 18	Precipitation of warmest quarter
BIO 19	Precipitation of coldest quarter

A numeric data matrix consisting of climate variables and altitude for each positive site was compiled and normalised using feature scaling. A dissimilarity matrix between each pair of observations was computed using Euclidean distance on the scaled matrix. To define climate clusters, minimising the total within-cluster variation (within-cluster sum of squares [WCSS]), we used the *k*-means clustering methods [41]. The *k*-means methods is the most commonly used unsupervised machine learning algorithm for partitioning and needs a pre-specified number of clusters [42]. Prior to applying the *k*-means clustering algorithm, we used two graph-based methods to determine the optimal number of clusters to use: (i) the elbow method and (ii) the silhouette method. The elbow method looks for the point at which the addition of a cluster does not result in any significant reduction in cluster variance, while the silhouette method evaluates the degree of separateness and cohesivity of the clusters [43].

The *k*-mean clustering method was performed using the number of clusters indicated by elbow and silhouette methods as initial centres, setting the maximum number of iterations and the random sets to 10,000.The dissimilarity matrix and the climate clusters were visualised, respectively, using R and QGIS software [44].

#### Phenological metrics

Phenological metrics were calculated for each climate cluster applying the method proposed by Edwards and Crone [45] already implemented in the study of arthropod seasonal activity [46, 47]. To compare the different phenological patterns of the climate clusters, we used the total abundance of *Phlebotomus* spp., collected on each sampling session, as the response variable. Abundance was log transformed to mitigate the influence of outliers. Gaussian models were fitted using generalised linear models with a negative binomial and Poisson distribution, both with the log link function, with day of year (DOY) and DOY^2^ as independent variables. To account for the interaction between DOY and climate cluster, the latter was added as a categorical factor in the model formulae. The selection of the best-fitting model was based on the second-order Akaike information criterion (AICc) value, calculating the ΔAICc between models [48, 49].

The coefficients of the best-fitting model (intercept and linear and quadratic terms) were used to calculate the phenological metrics for each climate cluster, following the procedure reported by Edwards and Crone [45]. The main phenological metrics were: DOY when the peak of adult sand fly abundance occurred (*μ*); standard deviation for the Gaussian distribution (*σ*); abundance (*N*), reported as percentage over the total predicted number of *Phlebotomus* spp.; and estimated number of individuals collected the day of peak abundance (*h*). The 95% confidence intervals and standard errors were calculated using parametric bootstrapping and delta method, respectively, with the ‘MASS’ and the ‘msm’ R packages for the *μ, σ* and *h* metrics [50–52]. The time interval within which 80% of sand flies were collected (80EA) was calculated as the number of days between the 10th and 90th percentile. The observable activity period was calculated as the number of days when the predicted response variable was > 1 [46].

In addition, the first and last DOY of activity were determined, respectively, as the day when number of *Phlebotomus* spp. exceeded zero or dropped to zero, according to the predicted values from the best-fitting Gaussian model.

#### Leishmania infection rate

A total of 228 pools from 36 sampling sites, located in nine provinces, were analysed (Table 2). At four sites, only 18 *Phlebotomus* specimens were captured; due to the limited number of female individuals, these samples were excluded from subsequent molecular analyses. Pools were defined according to the collection date and capture event (i.e. the individual trapping occasion) in order to preserve spatial and temporal resolution.

**Table 2:** Sites found to be positive for the presence of Phlebotominae, with relative species abundance (where species identification was carried out) and total number of collected sand fliesTable 2. Kindly check change to text in some header cells and to footnotes. If changes are incorrect, kindly modify on proofDone, thank you.

Climate cluster	Province	Code for sampling site	Latitude (°)	Longitude (°)	*Phlebotomus perniciosus* abundance (% of collected specimens)	*Phlebotomus perfiliewi* abundance (% of collected specimens)	Total number of Phlebotominae collected (*N* identified)	*Leishmania infatum* detection^a^	Positive pools % (*N* total pools) [mean* N* of Phlebotominae per pool]^a^	Minimum infection rate^a^	Maximum likelihood estimate^a^
1	Pisa	PI_2	43.74	10.33	50.00	50.00	12 (4)	NA	NA	NA	NA
	Livorno	LI_1	43.01	10.64	52.50	47.50	261 (20)	Positive	20.00 (5) [41.6]	0.69	0.53
	Grosseto	GR_1	42.77	10.90	50.00	50.00	16 (2)	Negative	0.00 (1) [14]	0.00	0.00
		GR_4	42.64	11.13	33.33	66.67	20 (3)	Negative	0.00 (1) [17]	0.00	0.00
		GR_5	42.72	11.09	0.00	100.00	5 (1)	Negative	0.00 (1) [5]	0.00	0.00
		GR_6	42.50	11.52	36.00	64.00	584 (36)	Positive	60.00 (5) [107.5]	0.89	0.85
		GR_7b	42.54	11.24	0.00	100.00	25,617 (51)	Positive	27.27 (33) [776.15]	0.04	0.36
		GR_8b	42.91	10.82	26.09	73.91	3025 (62)	Negative	0.00 (12) [243.33]	0.00	0.00
		GR_9	42.41	11.38	0.00	100.00	27 (3)	Negative	0.00 (1) [21]	0.00	0.00
		GR_10	42.48	11.21	50.00	50.00	69 (4)	Negative	0.00 (1) [65]	0.00	0.00
		GR_11	42.82	11.06	0.00	100.00	86 (12)	Negative	0.00 (4) [39.25]	0.00	0.00
	Viterbo	VT_2b	42.44	11.54	2.75	97.25	42,803 (72)	Positive	40.63 (64) [668.62]	0.07	0.84
		VT_4	42.25	11.71	58.93	41.07	139 (11)	Negative	0.00 (3) [56.66]	0.00	0.00
	Rome	RM_4	41.68	12.60	NA	NA	5 (0)	Negative	0.00 (1) [5]	0.00	0.00
2	Firenze	FI_3	43.73	10.79	100.00	0.00	26 (8)	Negative	0.00 (2) [8]	0.00	0.00
	Pisa	PI_1	43.46	10.74	0.00	100.00	608 (26)	Negative	0.00 (5) [109.2]	0.00	0.00
		PI_3	43.69	10.54	78.33	21.67	483 (20)	Negative	0.00 (5) [13.2]	0.00	0.00
		PI_4	43.38	10.93	9.71	90.29	3,113 (37)	Negative	0.00 (7) [425]	0.00	0.00
	Arezzo	AR_1	43.46	11.78	100.00	0.00	97 (30)	Negative	0.00 (3) [22.33]	0.00	0.00
	Siena	SI_1	43.29	11.48	3.24	96.76	1,569 (66)	Negative	0.00 (6) [177.66]	0.00	0.00
		SI_2b	43.12	11.49	1.43	98.57	1076 (66)	Negative	0.00 (7) [103.85]	0.00	0.00
		SI_3	43.42	11.27	0.00	100.00	735 (39)	Negative	0.00 (5) [124.6]	0.00	0.00
		SI_4	43.19	11.12	81.82	18.18	15 (11)	Negative	0.00 (1) [5]	0.00	0.00
		SI_5	43.27	11.33	0.00	100.00	198 (24)	Positive	33.33 (3) [50.33]	0.56	0.80
		SI_6b	42.99	11.76	0.00	100.00	983 (48)	Negative	0.00 (5) [163.4]	0.00	0.00
	Grosseto	GR_2	42.95	11.42	42.86	57.14	56 (12)	Negative	0.00 (3) [14.66]	0.00	0.00
		GR_3	42.94	11.29	50.00	50.00	7 (2)	Negative	0.00 (1) [5]	0.00	0.00
	Viterbo	VT_1	42.36	12.36	NA	NA	1 (0)	NA	NA	NA	NA
		VT_5b	42.37	11.87	1.43	98.57	13,925 (79)	Positive	38.10 (21) [642.85]	0.15	0.37
	Rome	RM_1	42.12	12.15	100.00	0.00	6 (3)	Negative	0.00 (1) [3]	0.00	0.00
		RM_2	42.13	12.08	37.50	62.50	49 (16)	Negative	0.00 (2) [16]	0.00	0.00
		RM_3	41.85	12.53	100.00	0.00	2 (1)	NA	NA	NA	NA
		RM_5	41.92	12.67	100.00	0.00	13 (1)	Negative	0.00 (1) [12]	0.00	0.00
		RM_6	41.89	12.85	77.11	22.89	156 (42)	Positive	60.00 (5) [21.2]	3.23	4.23
		RM_7	41.88	12.58	69.44	30.56	22 (10)	Negative	0.00 (4) [5.25]	0.00	0.00
3	Prato	PO_1	43.94	11.10	100.00	0.00	13 (2)	Negative	0.00 (1) [10]	0.00	0.00
	Firenze	FI_1	43.73	11.31	26.67	73.33	54 (8)	Negative	0.00 (3) [9.33]	0.00	0.00
		FI_2	43.82	11.26	52.38	47.62	82 (14)	Positive	50.00 (4) [10]	9.38	6.70
		FI_4	43.98	11.37	100.00	0.00	3 (3)	NA	NA	NA	NA
	Viterbo	VT_3	42.54	12.00	100.00	0.00	23 (10)	Negative	0.00 (1) [5]	0.00	0.00

*NA* Data not available

^a^The PCR results for *Leishmania infantum* detection were used to determine the occurrence of *L. infantum* in at least one tested pool, the percentage of positive pools and the average minimum infection rate and maximum likelihood estimation (MLE) for each site

^b^At these sites, in at least one event (abundant catch event), the total number of sand flies was estimated by weighing the collected sand flies

The minimum infection rate (MIR) and maximum likelihood estimate (MLE) were calculated for each capture event (combination site × date) in which at least three females were collected. MIR and MLE were calculated using the following formulae: $[\left( {{\text{number of positive pools}}/{\text{total specimens tested}}} \right)\, \times \,{1}00]$ for MIR and $\left\{ {[1 - (1 - Y/X)^{(1/m)} ]\,\, \times \,\,100} \right\}$ for MLE, where Y is the number of positive pools, X is the number of pools, and m is the pool size (number of sand flies in the pool). While MIR provides a conservative estimate of infection prevalence, MLE, accounting for pool size and number of tested pool, yields a more robust estimate of infection prevalence [53].

For the 14 abundant catches mentioned above, when males and females were pooled, the calculated sex ratio was used to estimate the total number of females examined.

MIR and MLE averages were calculated for each site.

## Results

### General outcomes

A total of 967 catches, accounting for a total catch of approximately 96,333 sand flies, were carried out in 13 provinces of the Tuscany and Lazio regions of Italy; of these, 131 catches were positive for the presence of *Phlebotomus* sp. Among the 78 sampling sites in total, 40 sites in nine provinces were positive for the presence of *Phlebotomus* spp. (Fig. 1b; Table 2). The 859 identified sand fly specimens belonged to two species: *P. perniciosus* (24%) and *P. perfiliewi* (76%). The highest abundances of sand flies (> 20,000) were recorded at two sites in two adjacent provinces of the southern Tuscany and northern Lazio regions (Grosseto and Viterbo), where *P. perfiliewi* was more abundant than *P. perniciosus* (Table 2). *Phlebotomus perniciosus* was never the dominant species at sites where more than 150 sand flies were collected, with the exception of PI_3 (Table 2), where approximately 500 sand flies were captured, of which about 80% were *P. perniciosus*.

**Fig. 2:**
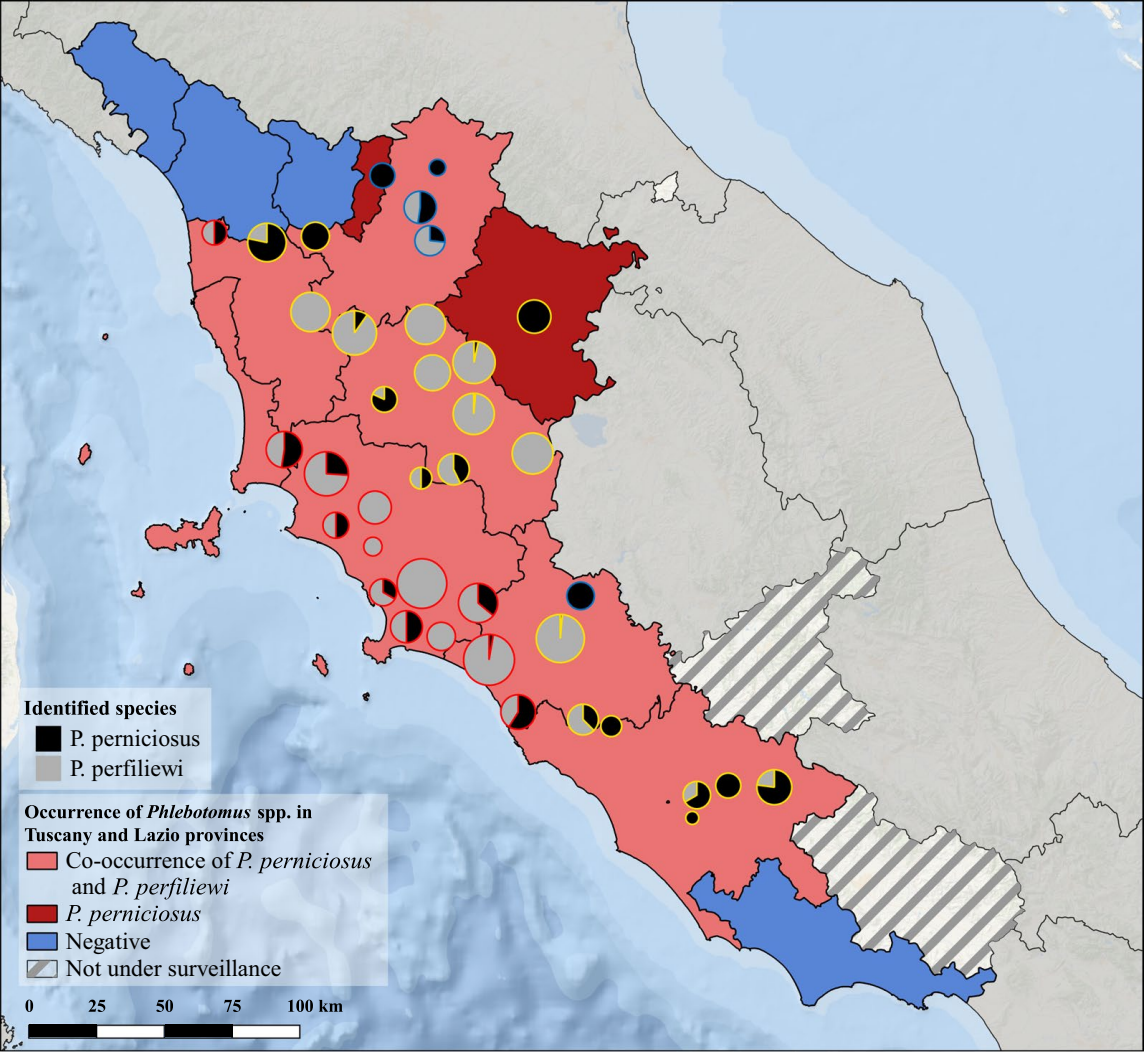
Distribution of *Phlebotomus* species according to their relative abundance. Cluster 1 is shown in red; cluster 2, in yellow; and cluster 3, in blue. The size of the pie chart is proportional to the total number of sand flies collected in each site. Provinces are coloured according to the presence, absence or co-occurrence of *Phlebotomus perniciosus* and *Phlebotomus perfiliewi*

With the exception of the four provinces that tested negative for the presence of sand flies, *P. perniciosus* and *P. perfiliewi* were found to co-occur in most of the geographical area included in the present surveillance study (Fig. 2). Eight sites were found to harbour exclusively *P. perniciosus*, and eight sites were found to harbour exclusively *P. perfiliewi*. The distribution and relative abundance of the two species are reported in terms of pooling the data by sampling site (Fig. 2; Table 2).

### Climate clusters

**Fig. 3:**
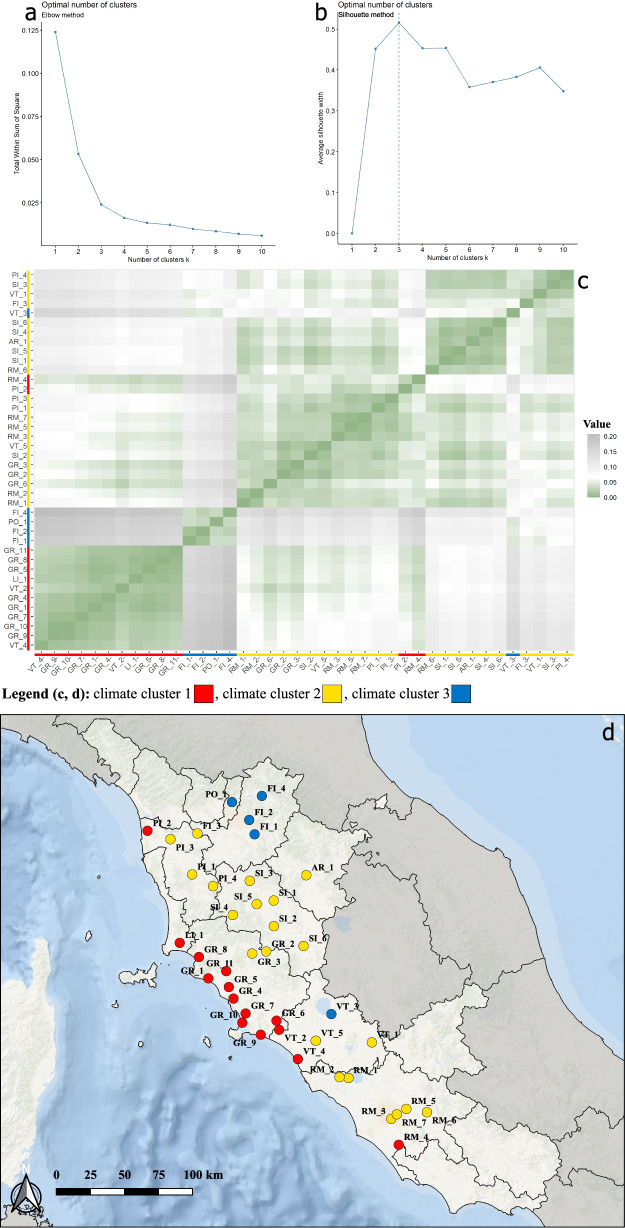
Graphical representation of the results of the elbow (**a**) and silhouette (**b**) methods. **c** Distance matrix showing the dissimilarity of each sampling site with respect to the other sites. The colour scale corresponds to the dissimilarity level. **d** Spatial distribution of all positive sampling sites for *Phlebotomus* spp. presence

The elbow and average silhouette methods, performed on the scaled matrix compiled with the 19 WorldClim variables (plus altitude), indicated that the optimal number of clusters was three (Fig. 3a, b). The *k*-mean method identified three climate clusters, referred to as clusters 1 (the more coastal one), 2 and 3 (the more inland one) hereafter; clusters 1, 2 and 3 had 14, 21 and 5 sites, respectively (Fig. 3c, d), and were characterised by WCSS values of 0.015, 0.006 and 0.003, respectively. Altitude differed among the three clusters; in particular, the sites within cluster 1, situated along the coastline, were characterised by the lowest altitude range (1–203 m a.s.l.).

**Fig. 4:**
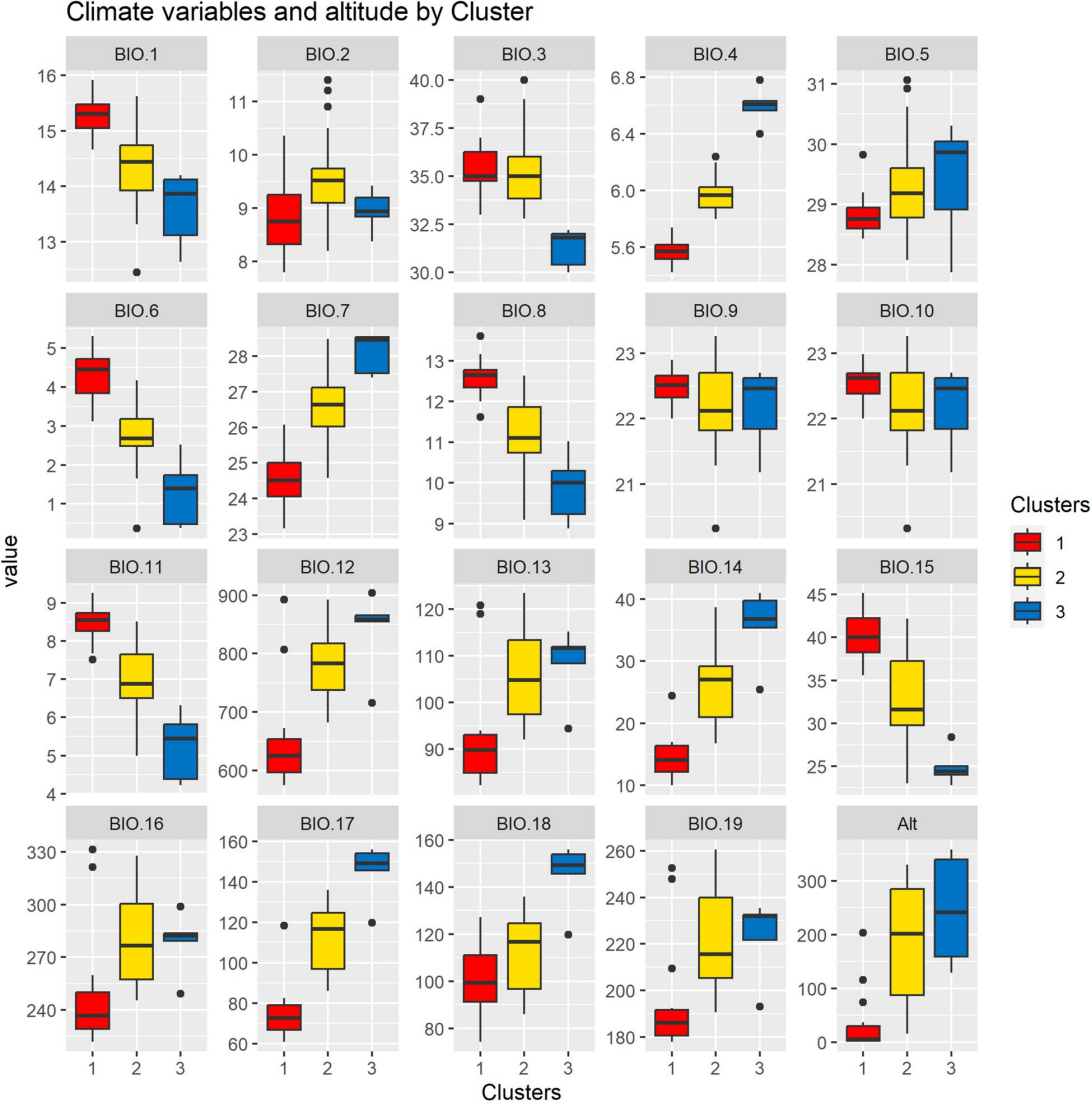
Boxplots of the WorldClim variables (plus altitude) for the three climate clusters. Cluster 1 is shown in red; cluster 2, in yellow; and cluster 3, in blue. See Table 1 and https://www.worldclim.org/data/bioclim.html for a description of the climate (BIO) variables

Although the average summer temperature was similar between the clusters (BIO 10), differences were recorded in the minimum temperature of the coldest month and in the average winter temperature (BIO 6 and BIO 11), which were higher for the first and second climate clusters. With respect to BIO 4 and BIO 7, which represent measures of annual temperature variation, the lowest values were recorded within the climate cluster 1, which showed a more constant climate, with a minimum temperature range of 3.1–5.3 °C in the coldest month and a maximum temperature range of 28.4–29.8 °C in the warmest month (Fig. 4). On the contrary, precipitation seasonality exhibited an opposite trend (BIO 15), with the highest annual variation, namely a strong difference in the rainfall regime between the wettest and driest periods of the year, reported in the first cluster (Fig. 4).

### Phenological metrics

The results of the phenological analysis revealed a higher deviance for the Gaussian model with the Poisson distribution compared to the Gaussian model with a negative binomial (Gaussian_Poisson_: null deviance = 1430.12 with* df* = 396, residual deviance = 960.39 with* df* = 387; Gaussian_negative_binomial_: null deviance = 438.34 with* df* = 396, residual deviance = 284.89 with* df* = 387). The Gaussian model with a negative binomial distribution showed a higher goodness of fit when the AICc of the respective models was compared (Gaussian_Poisson_ = 1,402.02, Gaussian_negative_binomial_ = 1,076.25, with |ΔAICc|= 325.77). The phenological metrics reported in Table 3 were then calculated for each climate cluster using the coefficients from the Gaussian_negative_binomial_ model.

**Table 3:** Summary of the results of the Gaussian models reporting the phenological metrics of *Phlebotomus* spp. activity for each climate cluster

Climate cluster	DOY peak (date in 2023)^a^		SD of activity^b^		Peak abundance^c^		*Phlebotomus* spp. abundance (%)^d^	80EA (*n* days)^e^	Observable activity period^f^	First DOY^g^	Last DOY^h^
	*μ*	95% CI pb (SE dm)	*σ*	95% CI pb (SE dm)	*h*	95% CI pb (SE dm)					
Cluster 1	218 (6 August)	211–224 (± 3)	26	23–31 (± 2)	6	4–9 (± 1)	48.24%	66	96	170	266
Cluster 2	225 (13 August)	220–231 (± 3)	27	24–32 (± 2)	4	3–6 (± 1)	35.90%	70	90	180	270
Cluster 3	216 (4 August)	164–245 (± 11)	36	25–88 (± 9)	1	1–3 (± 1)	15.86%	93	55	189	244

For the metrics a–c, the 95% confidence interval (CI) and standard error (SE) are reported. The parametric bootstrapping approach (pb) used the variance–covariance matrix of the coefficient estimates from the fitted Gaussian models with 10,000 simulated coefficients. The symmetrical SE was calculated according to the delta method (dm)

*80EA* Time interval within which 80% of sand flies were collected,* DOY* day of year,* SD* standard deviation

^a^DOY when the peak of *Phlebotomus* spp. abundance occurred (*µ*)

^b^SD for the Gaussian distribution (*σ*)

^c^*Phlebotomus* spp. abundance (*N*), reported as the percentage for each climate cluster over the total estimated abundance, where* h* is the estimated number of individuals collected the day of peak abundance

^d^Estimated peak of daily captured *Phlebotomus* spp. individuals, reported as the ratio in relation to the cluster with the minimum value (cluster 3)

^e^80EA duration, in days

^f^The number of days when the response variable is > 1: namely the natural logarithm of (*Phlebotomus* spp. abundance + 1)

^g^The predicted day of sand fly activity onset

^h^The predicted day when sand flies activity stopped

**Fig. 5:**
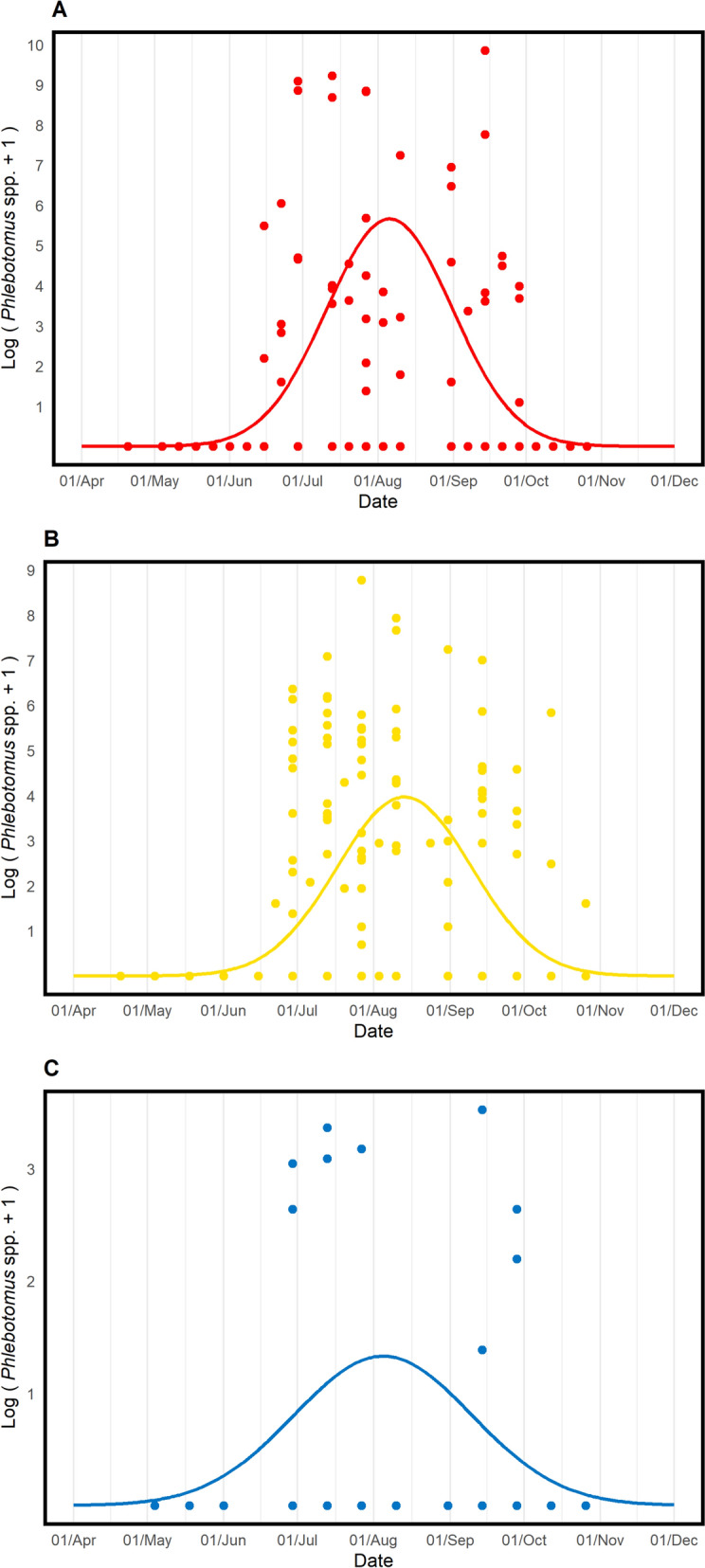
Gaussian model fitted to the seasonal trend of sand flies abundance, log transformed for each climate cluster. Seasonal trends were computed according to the results of the best-fitting Gaussian model (negative binomial) for cluster 1 in red (**a**), cluster 2 in yellow (**b**) and cluster 3 in blue (**c**)

The peaks of sand flies activity occurred between the 31 st and the 33rd epidemiological weeks of 2023 in all clusters, corresponding to the first weeks of August, with a mean standard deviation (*σ*) of 30 days. Sites within the first two clusters are characterised by higher abundance, with over 80% of sand flies expected to be detected; indeed, the Gaussian_negative_binomial_ model predictions indicated that approximately 50% and approximately 36% of sand flies were expected at sites within cluster 1 and cluster 2, respectively. The predicted peaks of abundance were six- and four-fold higher in cluster 1 and 2, respectively, than in cluster 3 (Table 3; Fig. 5). Given the correlation between the observable activity period and sand fly abundance [46], the number of days when the predicted number of captured sand flies is > 1 decreased from cluster 1 (96) to cluster 3 (55). The time interval of 80EA had a mean of 76 days (± 5).

The high abundance of sand flies predicted for clusters 1 and 2 is associated with an early onset of adult activity and a later end of this activity, compared to cluster 3 (Table 3). In terms of the relative abundance of *P. perfiliewi* and *P. perniciosus*, these two species were dominant in the first and third climate clusters, respectively, with sites characterised by the presence of one species only. Regarding the second climate cluster, at nine sites only one of the two species was identified: *P. perniciosus* in five sites in the provinces of Firenze, Arezzo and Rome; *P. perfiliewi* in four sites in the provinces of Pisa and Siena (Table 2). *Phlebotomus perfiliewi* was abundant in the first and second climate clusters, which were characterised by an altitudinal range of 0–300 m a.s.l., mild winters (minimum January temperature of 2–5 °C, BIO 6) and low summer precipitation (8–13 cm, BIO 18) (Fig. 4). *Phlebotomus perniciosus* was the most abundant species in cluster 3, which is characterised by cooler, wetter, high-altitude Mediterranean climates with strong seasonal temperature variability and pronounced winter precipitation. In addition, compared to cluster 3, clusters 1 and 2 were characterised by a smaller temperature variation over the year (BIO 4 and BIO 7) and a higher seasonality of rainfall (BIO 15) (Fig. 4).

### *Leishmania infantum* infection rate

**Fig. 6:**
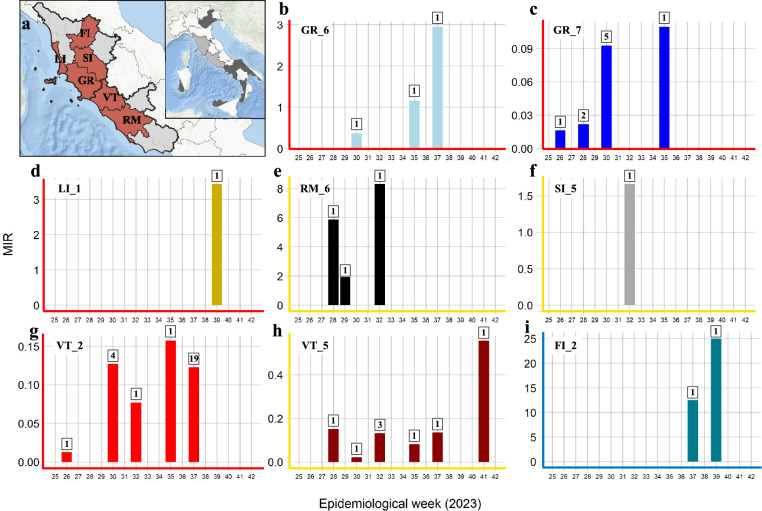
**a** Map of Tuscany and Lazio regions, with the six provinces where *Leishmania infantum*-infected sand flies were recorded highlighted in red. **b**–**i** Seasonal trend of minimum infectious rate (MIR) for the eight positive sites. Numbers above the bars indicate the number of positive pools, and each site is represented by bars of a different colour. The colours of the* x*- and* y*-axes represent the climate cluster to which they belong (cluster 1 in red, cluster 2 in yellow and cluster 3 in blue). The location and coordinates of these sites are given in Fig. 3 and Table 2. FL, Firenze; GR, Grosseto; LI, Livorno; RM, Rome; SI, Siena; VT, Viterbo

*Leishmania infantum* was detected in eight sites in six provinces (Rome, Viterbo, Grosseto, Siena, Livorno and Firenze) of the two regions under study (Fig. 6a). In these sites, 71 catches were carried out, of which 44 were positive for sand fly presence, with approximately 83,792 individuals captured, of which 78.09% (approx. 65,431) were females. Female sand flies were divided into 140 pools, with 53 of these pools testing positive for *L. infantum*.

Sorting the positive pools by catch and site, the MIR was calculated for 25 positive catches and the seasonal fluctuation is shown, by epidemiological week, in Fig. 6b–i. The first pools testing positive were catches from the 26th epidemiological week (end of June), from two sites located in bordering provinces, Grosseto and Viterbo, which also reported the highest number of positive pools per catch event (Fig. 6c, g) and the highest sand fly abundance (Table 2).

In terms of species identification, *P. perfiliewi* was the dominant species at these sites, with 97% of individuals in VT_2 and 100% in GR_7. A second site in the province of Viterbo (VT_5, Fig. 6h), which showed a high relative abundance of *P. perfiliewi* (99%), reported the highest number of positive catches (*N* = 6) and the longest time interval for the presence of infected sand flies, 14 weeks, with the last positive catch at epidemiological week 41 (mid-October).

Considering the eight sites where *L. infantum* infected sand flies were detected, four belonged to the first climatic cluster, three to the second and one to the third (Fig. 6b–i). The highest weekly MIRs were recorded in a site within the climate cluster 3 (Firenze province), where two pools of eight and four females, from 37th to 39th epidemiological week, tested positive (Fig. 6i). Considering the first and second climate clusters, the highest weekly MIRs were reported by two sites in the provinces of Grosseto and Livorno (approx. 3%; Fig. 6b and d) and one site in the province of Rome (approx. 8%; Fig. 6e).

## Discussion

The data reported here are the first to be derived from an extended surveillance of sand fly activity carried out over a large part of the territory of the Tuscany and Lazio regions. Our data confirm that *P. perfiliewi* and *P. perniciosus* are the dominant species and that they are found in sympatry at most of sites, co-occurring in seven out of the nine provinces where sand flies were found. Although found at different abundances, previous studies reported the detection of these two species at the same sites and in the same period, in central and southern Italy [29, 54].

*Phlebotomus perfiliewi* was the dominant species at the sites where the most abundant catches were recorded, located in central–southern Tuscany and northern Lazio (climate cluster 1), confirming previous reports from southern Tuscany [29, 55].

Our results confirm the high abundance and dominance of *P. perfiliewi* in rural environments [56], with catches at times exceeding 10,000 sand flies per night. Regarding *P. perniciosus*, our results confirm its ecological preference for hilly sites, characterised by moderate rainfall and temperate climate (climate cluster 3) [27, 57].

As all sand flies were sampled using the West Nile surveillance network, all sampling sites were set up in farms in rural and suburban areas, with two exceptions: sites within the urban area of Rome, where *P. perniciosus* was the dominant species, confirming the species’ adaptation to urban and peri-urban areas and previous data for Rome [58–60]. *Phlebotomus perniciosus* was also found to be dominant in rural domestic and peridomestic environments, confirming the ecological plasticity of the species [56].

One limitation of this study is that species identification was performed only on male sand flies. Female specimens require longer clarification and preparation procedures to allow for reliable morphological examination of diagnostic structures, particularly spermathecae and pharyngeal armour [61]. Since the main objective of this investigation was to explore the epidemiological context of sand fly occurrence, rather than to provide a detailed taxonomic assessment of species composition, the identification process prioritised male specimens, which can be distinguished more quickly than females. However, this approach may underestimate the full diversity of species present in the sampled populations. To mitigate this limitation, our interpretation relied on the extensive literature available for central Italy, which consistently reports the same sand fly species detected in the present study. Therefore, the male species composition observed in our study was used as a proxy to infer the likely composition of the female population. While this assumption is supported by previous regional studies [27, 29, 62], the absence of systematic female identification remains a methodological constraint. Future studies aimed specifically at characterising species diversity or assessing vector competence should include comprehensive morphological or molecular identification of both sexes to provide a more complete representation of the local sand fly community.

Considering the phenological analysis, over the entire territory surveyed, the peak of sand fly activity was recorded within the first 2 weeks of August, in accordance with previous findings from Italy [31, 54]. This period of high risk of vector–host contact, combined with increased tourist travel to Mediterranean countries in summer, could contribute to an expansion of *Leishmania* range. For example, new foci could be established when tourists return with their infected dogs to non-endemic EU countries [63, 64].

It is worth noting that we found differences in the duration of sand fly activity, with this duration being longer in the sites belonging to the first and second climate clusters and about half as long in the third cluster. The main climatic variables that may have influenced abundance and duration of sand fly adult activity between the different climate clusters are rainfall regime of the summer season and temperatures of winter and summer months. Indeed, as previously reported by observational and modelling studies [27, 57], high summer rainfall might reduce both the duration of the sand fly season as well as their abundance, while temperate climate may increase habitat suitability. In particular, higher winter temperatures and low summer precipitation associated with a more constant climate are coupled with higher sand fly abundance.

Regarding the peak of activity and sand fly abundance, the risk for vector–host contact would be higher in the first and second climate clusters. Notably, high numbers of *L. infantum*-positive catches were observed in Grosseto and Viterbo provinces, where the period at risk of infection ranges from the end of June to the first week of October. In these provinces, *P. perfiliewi* might be considered to be the most likely vector to sustain the transmission cycle, given the low abundance of *P. perniciosus*, the primary and more competent vector of *L. infantum* in Italy [22]. Notably, considering the eight sites where *L. infantum* infection in sand flies was detected, *P. perfiliewi* was dominant in five sites in the provinces of Grosseto, Siena and Viterbo, and in two of these sites it was the only species identified. This result is in agreement with previous research in which a fundamental role of *P. perfiliewi* in the transmission of CL was hypothesised and a high *Leishmania* parasite load was detected in females of this species [11, 29].

While the abundance of *P. perfiliewi* and its implication in the transmission of *Leishmania* parasites may explain the cluster of human VL cases recorded in the province of Grosseto in 2022, the known higher competence of *P. perniciosus* may explain the presence of VL cases in the provinces of Rome and Firenze. In fact, in two sites where *P. perniciosus* was more abundant than *P. perfiliewi*, the highest MIRs were recorded, namely near a notified human VL case in 2023 and within the same municipality of seven 2022 cases.

## Conclusions

Surveillance data reported in the present study reveal that both *P. perniciosus* and *P. perfiliewi* are relevant vectors of *L. infantum* in central Italy, although there are limitations to the present study. On one hand *P. perniciosus* has a higher competence, as demonstrated by the high MIR values recorded, but a lower abundance in the Lazio and Tuscany regions; on the other hand, *P. perfiliewi*, although less competent, has a higher vectorial capacity due to its overwhelming abundance, playing a pivotal role in *L. infantum* circulation in some areas of central Italy.

In conclusion, the integration of the entomological and human surveillance data with the ecological data of the vectors allowed us to define the differences in the transmission cycle of *L. infantum* within the study area, highlighting the role of the species involved. The results obtained could be useful for targeting prevention and public awareness campaigns.

## Data Availability

The data that support the findings of this study are available from the corresponding author upon reasonable request.

## Abbreviations

80EA: Time interval within which 80% of sand flies were collected
AICc: Second-order Akaike information criterion
BIO: Bioclimatic variables
CanL: Canine leishmaniasis
CL: Cutaneous leishmaniasis
HL: Human leishmaniasis
MIR: Minimum infection rate
ML: Mucocutaneous leishmaniasis
MLE: Maximum likelihood estimate
NPA: National Plan for Prevention, Surveillance and Response to Arboviroses
VL: Visceral leishmaniasis
WCSS: Within-cluster sum of squares
